# Stability of standardized uptake values for quantitative bone SPECT for jawbone lesions: a single-center cross-sectional study

**DOI:** 10.1186/s12903-024-04067-2

**Published:** 2024-03-05

**Authors:** Hironobu Hata, Satoshi Shimomura, Kenji Imamachi, Jun Sato, Takuya Asaka, Kenji Hirata, Kyousuke Funayama, Yoichi Mori, Masashi Matsuzaka, Toshikazu Nambu, Yoshimasa Kitagawa

**Affiliations:** 1 Department of Dentistry and Oral Surgery, NHO Hokkaido Cancer Center, Sapporo, Japan; 2 Department of Radiology, NHO Hokkaido Cancer Center, Sapporo, Japan; 3 Oral Diagnosis and Medicine, Department of Oral Pathobiological Science, Faculty of Dental Medicine, Hokkaido University, Sapporo, Japan; 4 Department of Diagnostic Imaging, Graduate School of Medicine, Hokkaido University, Sapporo, Japan; 5 Clinical Research Support Centre, Hirosaki University Hospital, Hirosaki, Japan; 6 Department of Medical Informatics, Hirosaki University Hospital, Hirosaki, Japan; 7 Department of Dentistry and Oral Surgery, NHO Hokkaido Cancer Center, 3-54, Kikusui4-Jyo 2-Tyoume, Sapporo Shiroishi-Ku, Hokkaido, 003-0804 Japan

**Keywords:** SPECT, Quantitative analysis, MRONJ, Uptake time, Jawbone

## Abstract

**Background:**

The long time required for bone uptake of radiopharmaceutical material after injection for bone scintigraphy is a burden for patients with poor health. Thus, to assess whether the uptake time could be reduced for single-photon emission computed tomography (SPECT) of the jawbone, this study evaluated differences in maximum standardized uptake values (SUVmax) within patients using SPECT imaging at 2 and 3 hours after radiopharmaceutical injection.

**Methods:**

A total of 33 patients undergoing treatment or in post-treatment follow-up for medication-related osteonecrosis of the jaw, who visited our hospital between July 2020 and August 2021 and could receive SPECT twice on the same day, were enrolled in the study. Patients were injected with technetium-99 m hydroxymethylene diphosphonate (Tc-99 m HMDP) intravenously. The SUVmax for healthy parietal bones and jawbone lesions were calculated from the SPECT images using quantitative analysis software, and the SUVmax were compared between 2- and 3-hour uptake times.

**Results:**

After exclusion, 30 patients were included in the study. In the 2-hour and 3-hour images, the median SUVmax of the parietal bones were 1.90 and 1.81, respectively, and those of the jawbone lesions were 9.25 and 9.39, respectively. The limits of agreement (LOA) ranged from − 0.33 to 0.25 in the parietal bones, and the %LOA ranged from − 9.8 to 17.3% in the jawbone lesions, showing high equivalence between the two uptake durations. The SUVmax showed no clinical differences between the 2- and 3-hour uptake durations for Tc-99 m HMDP SPECT of the jawbone.

**Conclusions:**

The results of this study justify a 2–3-hour uptake window when performing quantitative SPECT of the jawbone. Therefore, the minimum uptake time can potentially be reduced to only 2 hours.

## Background

Bone scintigraphy is a functional imaging procedure that employs nuclear medicine imaging techniques to assess the metabolic turnover of bone. For decades, this technique has been used in the diagnosis of many bone conditions, including bone cancer and metastases, inflammation, fractures, and infections. In recent years, it has also been used to assess medication-related osteonecrosis of the jaw (MRONJ). Historically, it has been difficult to assess the degree of inflammation in and the effects of anti-inflammatory therapy on MRONJ. Objective indexes, which could be used to evaluate the range and intensity of technetium-99 m (Tc-99 m) methylene diphosphonate (MDP)/hydroxymethylene diphosphonate (HMDP) accumulation in bones, are lacking. In recent years, advances in single-photon emission computed tomography (SPECT) devices, techniques such as computed tomography (CT)-based attenuation correction through in-device integration with CT, and software development have contributed to improved quantitative analysis. Current bone scintigraphy guidelines provide a wide range of permissible uptake durations for delayed-phase imaging (i.e., 2–4 h after injection of radiopharmaceutical material) [1, 2]. However, in the quantitative analysis of bone scintigraphy, it has not been confirmed whether the quantification is stable within the wide acceptable range presented by these guidelines.

At our hospital, we typically perform whole-body bone scans and bone SPECT image acquisition after a 3-h wait time, based on the principle that 2 h is insufficient for the clearance of the radiotracer from soft tissue. However, we propose that this window should be re-examined and reduced to the shortest clinically acceptable duration. This would significantly reduce the burden on patients who may be in poor physical condition. At the same time, a 2-h wait time would allow for an increased number of examinations per day, providing an advantage to hospital efficiency. Reducing the duration of hospital stays is also advantageous for patients who are visiting from afar as well as for preventing nosocomial infections. However, standardizing the quantitative evaluation of SPECT imaging procedures should be evaluated through rigorous research, as imaging time protocols are not currently unified or standardized across hospitals. Hence, we aimed to assess maximum standardized uptake values (SUVmax) for SPECT imaging following 2- and 3-h uptake durations. We hypothesized that it would be possible to reduce the uptake time to a minimum of 2 h when performing Tc-99 m HMDP quantitative SPECT of the jawbone.

## Methods

### Patients and study design

Thirty-three patients diagnosed with MRONJ or cured MRONJ in our dental and oral surgery department between July 2020 and August 2021 were enrolled in the study. The study followed an observational, cross-sectional, within-subjects design.

#### Inclusion criteria

The inclusion criteria were as follows: patients who (1) were diagnosed with MRONJ or were cured after MRONJ treatment, (2) had undergone bone SPECT to image the craniofacial region, (3) provided informed consent, (4) were older than 20 years of age, (5) had received cancer treatment with anti-resorptive drugs (ARDs), and (6) consented to undergo bone scans twice on the same day with scan acquisition start times of 2 and 3 h after injection of the radiopharmaceutical substance.

#### Exclusion criteria

The exclusion criteria prior to data analysis were as follows: patients (1) with high accumulations suggestive of parietal bone metastases on SPECT images, as the right and left parietal bones were treated as representative of healthy bone, or (2) without significant accumulation in the jaw lesions.

#### MRONJ classification

In line with the 2014 AAOMS position paper, MRONJ was classified according to the MRONJ staging system [3]. Patients with MRONJ at any disease stage and patients under follow-up care after successful treatment of MRONJ were included. Successful treatment of MRONJ (cured MRONJ) was considered when all symptoms and signs, including exposed bone, disappeared. The diagnosis of MRONJ and cured MRONJ was made by oral surgeons with more than 10 years of clinical experience.

#### Ethics approval and consent to participate

This cross-sectional study was conducted in accordance with the Declaration of Helsinki, and the protocol was approved by the Hokkaido Cancer Center Hospital Ethics Review Board (registration number: 02–31). Verbal informed consent was obtained from all participants, and consent acquisition was recorded in the patients’ medical records.

### Data acquisition

Bone SPECT imaging was performed after an intravenous injection of 555 MBq Tc-99 m HMDP (Clearbone® 99mTc-HMDP; Nihon Medi-Physics Co., Ltd., Tokyo, Japan) using a SPECT dual-head gamma camera system (Discovery NM630; GE Healthcare, Chicago, IL, USA). The SPECT images were acquired using the following parameters: a low-energy high-resolution collimator; step-and-shoot mode with 30 s per step and 72 steps per detector; 360-degree view; matrix size of 128 × 128; pixel size of 3.32 mm; and energy window of 140.5 keV ± 10%. The interval between the injection of Tc-99 m-HMDP and the start of acquisition of the first SPECT image was 2 h, and that for the second image was 3 h, which have been referred to as the 2-h and 3-h image uptake times, respectively. When there were multiple lesions in the jawbone, the site with the highest SUVmax was used as the representative value for the patient.

### Image reconstruction

The SPECT images were reconstructed using the ordered subset expectation maximization method, with 10 iterations and 10 subsets. The number of iterations and subsets was selected using phantom experiments so as to be reproducible and close to the theoretical values of SUV measurements. Images were smoothed with a three-dimensional spatial Gaussian filter (10 mm full width at half maximum). We applied resolution recovery to model the point spread function and corrected it for reconstruction. The Becquerel calibration factor (BCF), a numeric factor used to convert pixel values into SUVs, was measured using a cylindrical phantom filled with a uniform Tc-99 m solution. The BCF was determined at 8,700 Bq/counts/s. The SUVs were calculated as follows [2]: [BCF (Bq/cps) × body weight (g) × SPECT count density (count/cc)] / [scan duration (s) × injected activity (Bq)].

### Data analysis

Data were analyzed using GI-BONE, the bone SPECT quantitative analysis software included in the medical device software package AZE Virtual Place Hayabusa (ver. 9.0; Nihon Medi-Physics Co., Ltd.). More specifically, the software computed the SUVmax for the bilateral parietal bones and the osteomyelitis lesion in the jawbone by setting the volume of interest semi-automatically. The SUVmax represented a high inflammatory activity spot in the MRONJ module and were measured using pre-specified GI-BONE software volume of interest settings for the bilateral parietal bones. The SUVmax of the bilateral parietal bones as normal bones were calculated by setting the volume of interest within a 12-pixel cube (62.1 cm^3^) [4, 5]. The 2-h and 3-h image alignments were automatically coregulated by the software. SPECT image analysis using GI-BONE was performed by a dentist and a radiologist who were familiar with this software.

### Statistical analysis

The differences in the SUVmax between the 3-h and 2-h images were calculated at two sites (i.e., each patient’s parietal bones and jawbone lesions). The data were first screened for normal distribution using the Shapiro–Wilk test. Intra-examiner reliability was checked using intraclass correlation coefficients (ICCs). ICCs and Bland–Altman plots were used to analyze the data. The limits of agreement (LOAs) of the measurement methods were determined as the mean difference ± 1.96 × the standard deviation of the difference. All statistical analyses were performed using JMP® Pro 16.0 software (SAS Institute Inc., Cary, NC, USA).

## Results

### Patient characteristics

Among the 33 patients who underwent SPECT imaging twice in 1 day, two were excluded due to cranial bone metastases observed within the SPECT images and one due to a lack of significant accumulation in the jawbone. As a result, 30 patients were included in this study. The enrolled patients included 10 men and 20 women with a median age (Quartile 1, Quartile 3) of 70.5 years (60.3, 73.0). Data from individual patients are presented in Table 1. The differences in SUVmax between the 2-h and 3-h images were normally distributed at both sites (Table 1).

**Table 1:** Characteristics of all the patients and SUVmax of the parietal bones and jawbone lesion

No.	Sex	Age (years)	Primary cancer disease	ARD	MRONJ stage	Jawbone	SUVmax of the parietal bones			SUVmax of the jawbone lesion		
							2 h	3 h	3 h − 2 h	2 h	3 h	3 h − 2 h
1	M	75	Multiple myeloma	ZA-Dmab	2	Maxilla and mandible	1.58	1.54	-0.04	17.21	17.32	0.11
2	F	65	Breast cancer	ZA-Dmab	1	Mandible	2.91	3.32	0.41	9.35	11.06	1.71
3	F	73	Breast cancer	ZA-Dmab	3	Mandible	2.49	2.51	0.01	7.21	6.65	-0.56
4	M	78	Prostate cancer	ZA	Healed	Mandible	1.11	0.86	-0.25	5.56	5.13	-0.43
5	M	78	Prostate cancer	Dmab	0	Mandible	1.46	1.42	-0.04	10.34	9.83	-0.51
6	M	80	Prostate cancer	ZA-Dmab	Healed	Maxilla	1.85	1.80	-0.04	5.64	6.82	1.18
7	F	55	Breast cancer	ZA-Dmab	2	Maxilla	1.64	1.67	0.03	11.38	11.81	0.43
8	F	57	Breast cancer	Dmab	2	Mandible	1.34	1.37	0.03	6.66	7.42	0.76
9	F	73	Breast cancer	Dmab	3	Maxilla	2.86	2.75	-0.11	10.46	12.12	1.66
10	F	73	Breast cancer	Dmab	2	Mandible	1.95	2.18	0.23	8.43	8.98	0.55
11	F	70	Lymphoma	Alendronate	2	Mandible	3.06	2.95	-0.12	9.27	9.42	0.15
12	F	73	Breast cancer	Dmab	2	Mandible	2.91	2.61	-0.30	16.93	18.13	1.20
13	F	70	Breast cancer	Dmab	1	Mandible	1.61	1.56	-0.05	4.88	4.64	-0.24
14	F	78	Breast cancer	Dmab	3	Maxilla	3.27	3.30	0.03	10.50	10.17	-0.33
15	M	61	Prostate cancer	Dmab	0	Mandible	1.61	1.60	-0.01	16.45	16.88	0.43
16	F	60	Breast cancer	Dmab	2	Mandible	1.96	1.78	-0.18	5.11	5.17	0.06
17	F	71	Breast cancer	Dmab	2	Maxilla	1.40	1.45	0.04	10.70	11.05	0.35
18	F	52	Breast cancer	Dmab	0	Maxilla	1.52	1.40	-0.12	5.05	5.38	0.33
19	M	73	Multiple myeloma	Dmab	1	Mandible	1.60	1.66	0.06	14.53	14.64	0.11
20	F	81	Multiple myeloma	Dmab	0	Mandible	2.59	2.67	0.08	6.54	6.53	-0.01
21	M	72	Lung cancer	Dmab	2	Mandible	1.47	1.30	-0.17	5.10	5.54	0.44
22	F	66	Prostate cancer	Dmab	2	Mandible	2.02	2.09	0.07	5.02	5.09	0.07
23	M	75	Thyroid cancer	ZA	2	Mandible	2.18	1.89	-0.29	11.69	12.93	1.24
24	F	56	Breast cancer	Dmab	2	Maxilla	1.75	1.82	0.07	9.22	9.36	0.14
25	F	69	Breast cancer	ZA-Dmab	Healed	Maxilla	2.39	2.44	0.05	6.18	6.94	0.76
26	F	59	Breast cancer	ZA-Dmab	2	Mandible	1.64	1.54	-0.10	6.48	6.10	-0.38
27	M	73	Multiple myeloma	Dmab	2	Mandible	1.40	1.37	-0.02	11.39	12.27	0.88
28	F	51	Breast cancer	ZA-Dmab	2	Mandible	2.15	2.00	-0.16	6.20	6.23	0.03
29	F	70	Breast cancer	Dmab	3	Maxilla	2.35	2.28	-0.07	11.11	11.75	0.64
30	M	58	Lung cancer	Dmab	2	Mandible	2.33	2.14	-0.19	10.46	11.26	0.80
Median		71					1.90	1.81	-0.04	9.25	9.39	0.34
Q1–Q3		(60.3–73.0)					(1.58–2.38)	(1.54–2.40)	(-0.12–0.04)	(6.19–11.01)	(6.31–11.80)	(0.04–0.76)

SUVmax, maximum standardized uptake value; ARD, anti-resorptive drug; MRONJ, medication-related osteonecrosis of the jaw; Q1, quartile 1; Q3, quartile 3; M, male; F, female; ZA, zoledoronate; Dmab, denosumab

**Fig. 1:**
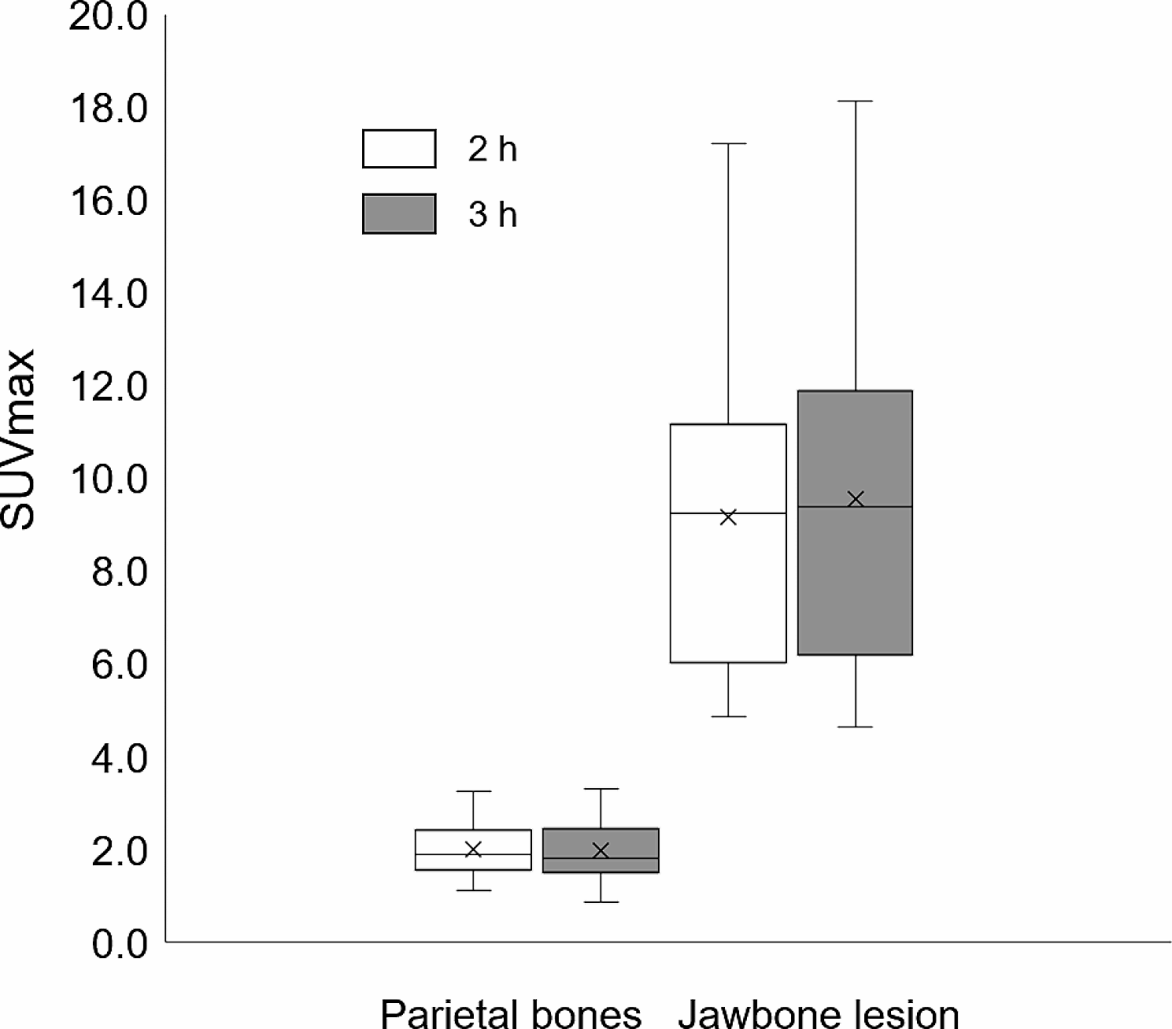
SUVmax for varying parietal bone SPECT imaging acquisition times. The median SUVmax (Quartile 1, Quartile 3) of the parietal bones is 1.90 (1.58, 2.38) and 1.81 (1.54, 2.40) for the 2-h and 3-h images, respectively, with a median difference of 0.9. The median SUVmax of the jawbone lesions is 9.25 (6.19, 11.01) and 9.39 (6.31, 11.80) for the 2-h and 3-h images, respectively, with a median difference of 0.14. *SUVmax*, maximum standardized uptake value; *SPECT*, single-photon emission computed tomography

The ICCs for the 2-h and 3-h SUVmax for the parietal bones and jawbone lesions showed high reproducibility, with ICC (1,1) values of 0.97 (R^2^ = 0.94) and 0.98 (R^2^ = 0.98), respectively (Fig. 1).

**Fig. 2:**
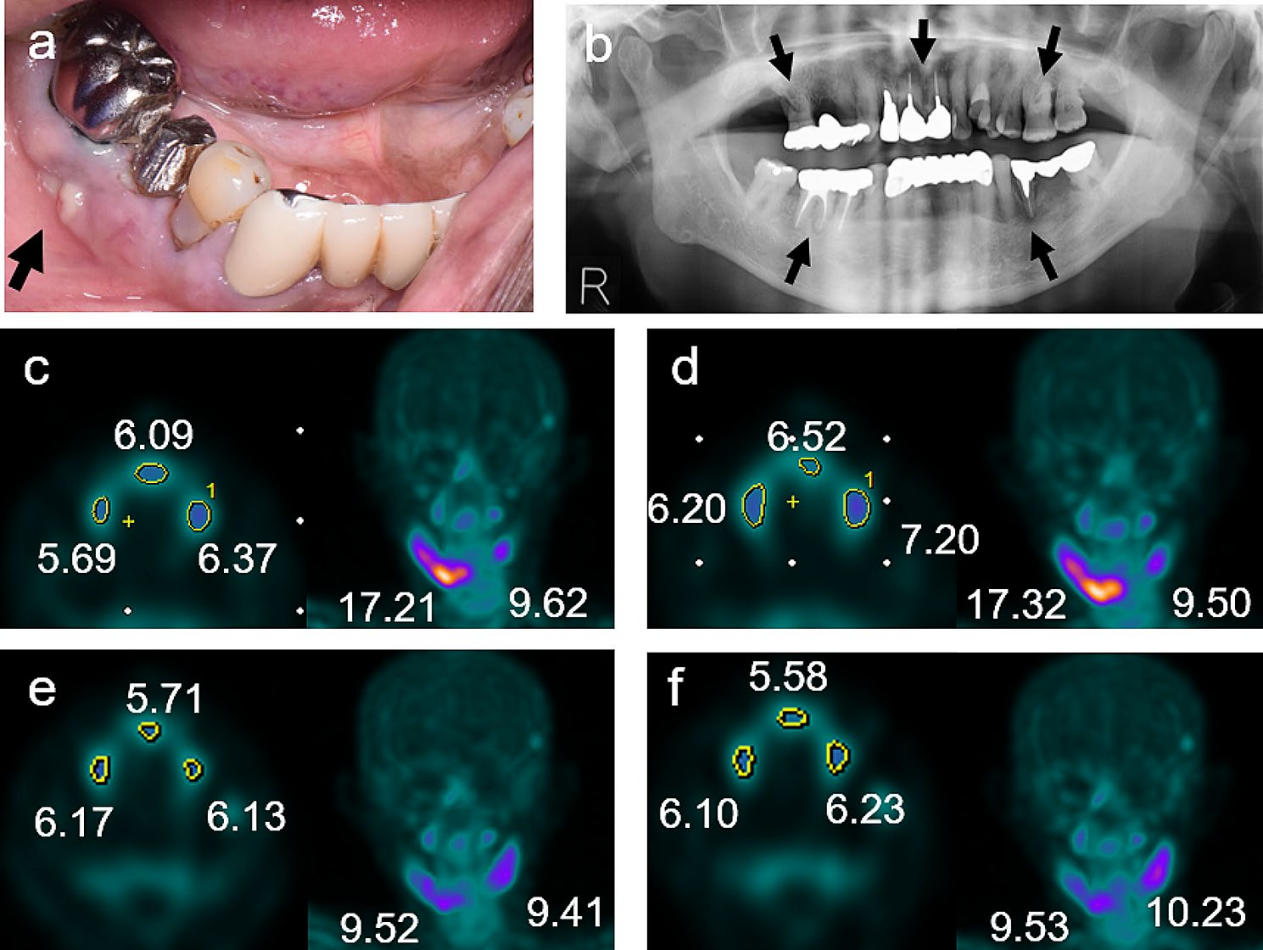
Depiction of a representative case (Patient 1). Patient 1 was a 75-year-old man who had been treated for multiple myeloma at our hospital. Zoledronate was administered four times, and denosumab was administered nine times. Swelling and drainage of pus from the fistula in the buccal gingiva of the second premolar of the right mandible (black arrow) (**a**). In the panoramic radiograph, periapical lesions are seen in five teeth (16, 21, 26, 35, and 45) (black arrows) (**b**). In the 2-h image from the first SPECT, strong accumulation in the right mandible, moderate accumulation in the left mandible, and weak accumulation in the maxilla are observed at three sites (**c**). There is an equivalence between the findings of the 3-h and 2-h images for the first SPECT (**d**). In the 2-h image for the second SPECT (conducted 3 months later), the strong accumulation in the right mandible has improved to moderate accumulation (**e**). There is an equivalence between the 3-h and 2-h images for the second SPECT (**f**). *SPECT*, single-photon emission computed tomography

Representative results acquired from Patient 1 are presented in Fig. 2. The MRONJ stage was determined to be stage 2, and initial bone SPECT at 2 h revealed an SUVmax of 17.21 for the right mandible, indicating severe inflammation. A moderate Tc-99 m HMDP accumulation (SUVmax, 9.62) was similarly observed in the left mandible, and a low accumulation (SUVmax, 5–6) was observed in the maxilla at the three sites (Fig. 2c). The sites with the strongest accumulation in the right mandible were evaluated as representative sites in this study and showed almost the same SUVmax in the 2-h (17.21) and the 3-h (17.32) images. Notably, Patient 1 presented with three weak accumulation sites in the maxilla and one moderate accumulation site in the left mandible, with all sites showing similar SUVmax values on the 2-h (5.69, 6.09, 6.37, and 9.62) and the 3-h (6.20, 6.52, 7.20, and 9.50) images (Fig. 2c and d).

**Fig. 3:**
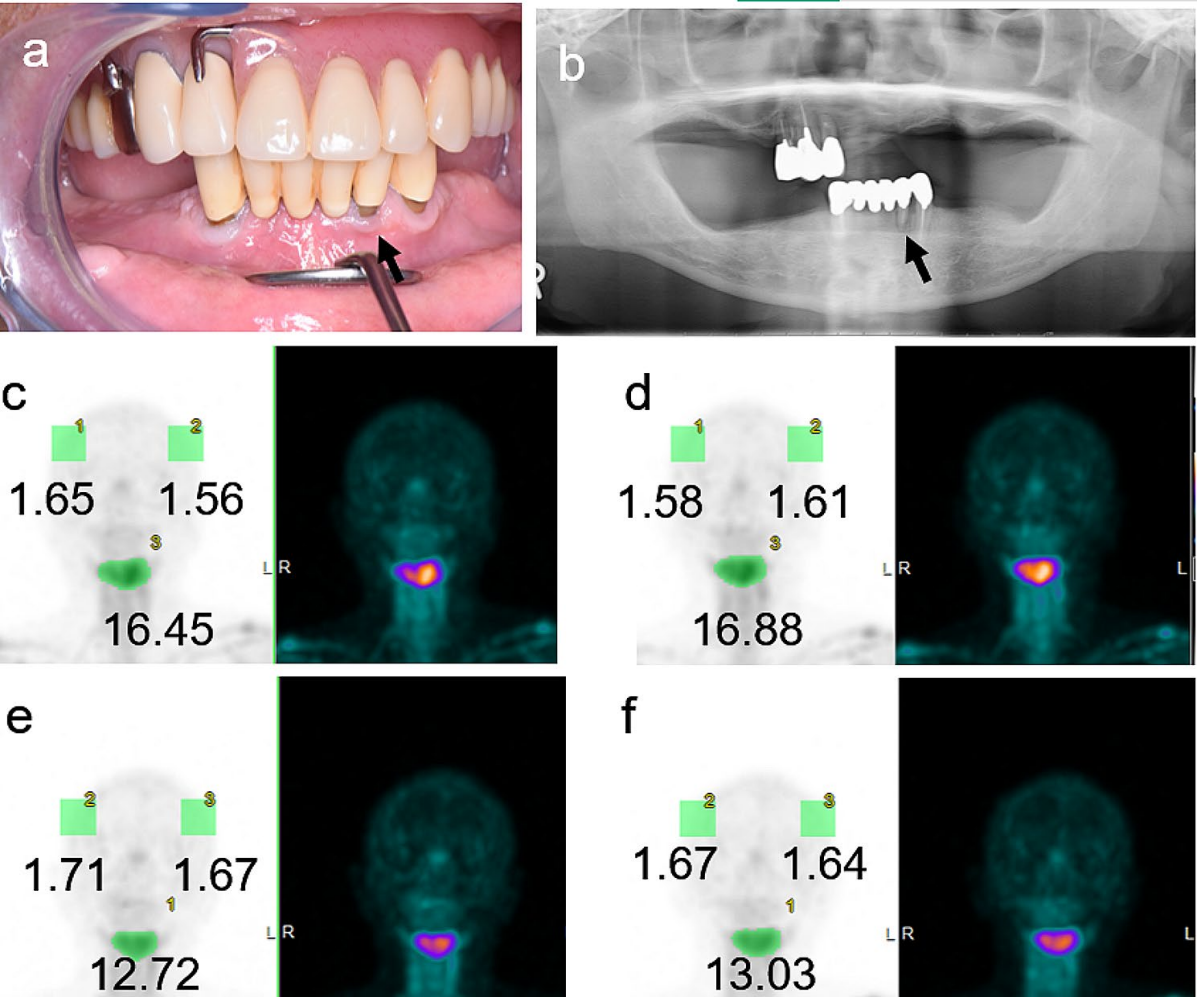
Depiction of a representative case (Patient 15). Patient 15 was a 61-year-old man who had been treated for bone metastasis of prostate cancer at our hospital. After being administered denosumab 15 times, the patient complained of mandibular pain while wearing a mandibular denture. No gingival swelling or bone exposure is observed in tooth number 32 (black arrow) (**a**). However, the tooth was tender on percussion. A panoramic radiograph shows a widening of the PDL space, suggestive of periodontitis, in tooth number 32 (black arrow) (**b**). The 2-h image for the first SPECT shows an SUVmax of 16.45 and strong accumulation in the midline of the mandible (**c**). In the 3-h image for the first SPECT, both the SUVmax of the parietal bones and SUVmax of the jawbone lesions are highly equivalent to the SUVmax of the corresponding 2-h images (**d**). In the second SPECT (2-h image), which was obtained 3 months later, improvement is observed in the lesion (SUVmax, 12.72). However, the SUVmax of the parietal bones has not changed (**e**). In the second SPECT, as in the first SPECT, the SUVmax of the parietal bones and the jawbone lesions show high equivalence in the comparative imaging evaluations of the 2-h and 3-h images (**f**). *PDL*, Periodontal ligament; *SPECT*, single-photon emission computed tomography; *SUVmax*, maximum standardized uptake value

Furthermore, representative results from Patient 15 are presented in Fig. 3. The patient was diagnosed with MRONJ stage 0. The 2-h bone SPECT image showed a strong accumulation (SUVmax, 16.45) in the midline of the mandible (Fig. 3c). The SUVmax at 2-h and 3-h imaging of the parietal bones and the jawbone lesions showed high equivalence (Fig. 3c and d).

### Follow-up SPECT

In these two representative cases, follow-up SPECT was possible after anti-inflammatory treatment, and the results are also presented below for further illustration. In Patient 1, the follow-up SPECT (conducted 3 months later) showed improvements from strong accumulation in the right mandible (SUVmax, approximately 17.0 on the 2-h and 3-h images) to moderate accumulation (SUVmax, approximately 9.5 on the 2-h and 3-h images). However, in the left mandible and the maxilla, little reduction in accumulation was observed from the initial SPECT images. The quantitative analysis of the follow-up SPECT showed that, as in the first images, the SUVmax of each accumulation site in the jawbone was almost the same in the 2-h and 3-h images (Fig. 2e and f).

Furthermore, in Patient 15, the SUVmax of the parietal bones was almost the same between the initial SPECT images and follow-up images after 3 months of anti-inflammatory treatment. However, the high accumulation intensity of the lesion’s SUVmax at approximately 16.5 on the initial SPECT 2-h and 3-h images improved to an SUVmax of approximately 13.0 on the follow-up SPECT 2-h and 3-h images, indicating a therapeutic anti-inflammatory effect (Fig. 3c, d, e, and f). In the quantitative analysis of the subsequent SPECT, similar to that in the initial SPECT, the SUVmax of the parietal bones and the jawbone lesions showed high equivalence between the 2-h and 3-h images (Fig. 3e and f). These findings were similar to those in Patient 1 and demonstrated high intra-patient reproducibility at two different time points.

### Bland–Altman analysis of the parietal bones and jawbone lesions

**Fig. 4:**
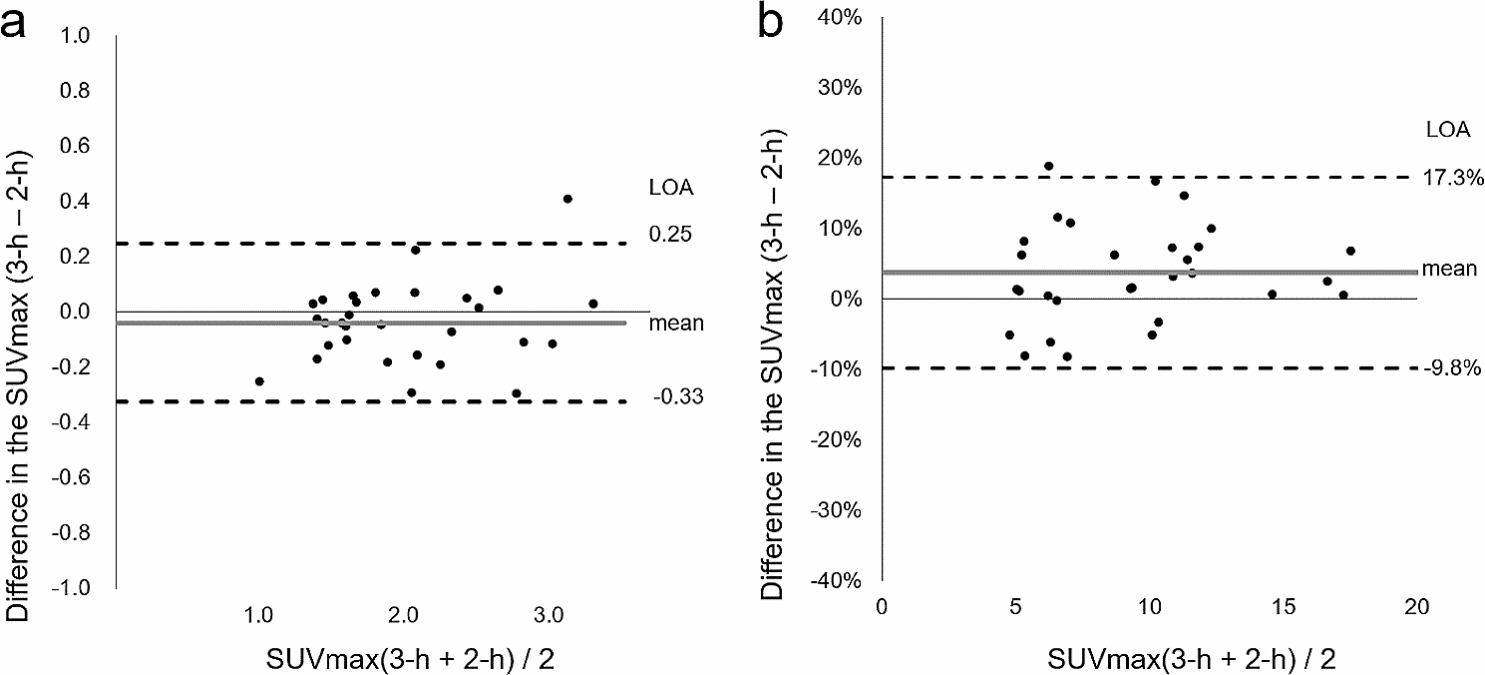
Comparison of the 2-h and 3-h images. (**a**) The permissible LOA for the difference in the SUVmax of the parietal bones (with no systematic error between the 2-h and 3-h images) ranges from − 0.33 to 0.25. (**b**) For the jawbone lesion, with systematic errors in the 2-h and 3-h images in terms of SUVmax, the LOA calculated by creating a percent difference plot ranges from − 9.8–17.3%. *LOA*, limit of agreement; *SUVmax*, maximum standardized uptake value

The average difference (95% confidence interval [CI]) between the SUVmax of the 2-h and 3-h images was − 0.04 (-0.092–0.017) for the parietal bones; the associated t-value was − 1.40. Since the 95% CI (i.e., -0.092–0.017) included zero, we judged that there was no fixed bias between the two imaging modalities at a statistical significance level of 5% with 28 degrees of freedom. The regression equations for the two indicators did not differ at the level of statistical significance, and there was no proportional bias. The calculated LOA ranged from − 0.33 to 0.25, and the LOA range was exceeded only in one patient. An extremely high equivalence was observed (Fig. 4a).

The average difference (95% CI) in the jawbone SUVmax when evaluating images of the lesion obtained after 2 and 3 h of uptake was 0.39 (0.158–0.61); the t-value was 3.46. Since the 95% CI (i.e., 0.158–0.61) of the mean value of the lesion did not include zero, we judged that there was a fixed bias between the 2-h and 3-h images at a statistical significance level of 5% with 28 degrees of freedom. The regression equations for the two indicators were significantly different, and we observed a proportional bias. Since there was a systematic bias in the difference in SUVmax at the lesion, we created a relative axis plot to eliminate this apparent systematic bias and calculate the LOA. The average difference in the SUVmax of the lesion was 3.8%, and the LOA ranged from − 9.8 to 17.3% (Fig. 4b). The difference between the two images was relatively minimal even at the site of the lesion; the LOA was within ± 20%, and a high equivalence was confirmed between the two images.

## Discussion

To the best of our knowledge, no prior study has evaluated the optimal uptake time for bone SPECT using a quantitative evaluation of the craniomaxillofacial bones. Our results suggested that the SUVmax obtained with a 2-h uptake duration was clinically equivalent to that obtained with a 3-h uptake duration when conducting Tc-99 m HMDP SPECT of the parietal bones and jawbone lesions, with extremely high reproducibility.

Of the three patients for whom follow-up imaging was obtained after anti-inflammatory treatment, the cases of two patients in whom the treatment effects were confirmed (i.e., SUVs at representative sites were reduced) were presented herein as representative cases showing the intra-patient reproducibility of 2-h and 3-h images. Both cases showed high intra-patient reproducibility at two different time points, which validated our results from another aspect.

Accurately selecting the optimal uptake duration is essential for obtaining the optimal image quality and can affect diagnoses and recommended courses of treatment. The current guidelines for bone scintigraphy recommend that bone phase imaging should range from 2 to 4 h after the intravenous administration of radiopharmaceuticals [1, 6]. However, this recommended time range is wide, and imaging times currently vary across facilities. Tc-99 m-labeled diphosphonate allows for clearance of the radiotracer from soft tissues during the uptake time, thus increasing the target-to-background ratio and improving bone visualization [7, 8]. The injected radiolabeled bisphosphonates are adsorbed onto the surface of hydroxyapatite crystals proportionally to local osteo-angiogenesis and osteoblast activity.

After intravenous administration, the plasma clearance of bisphosphonates is biexponential and is a function of skeletal uptake and excretion. Four hours after injection, approximately 50–60% of the injection volume is fixed to the skeleton. The unbound fraction (34%) is excreted in the urine, and approximately 6% remains in circulation. Maximum bone accumulation is reached 1 h after tracer injection and remains constant for up to 72 h [1]. Our results showed high equivalence between SUVmax values when conducting comparative evaluations of 2-h and 3-h images. The jawbone is close to the surface of the body; hence, the SUVmax appeared to be unaffected by background signals due to the pharmacokinetics of Tc-99 m-labeled diphosphonate clearance.

The Bone Scan Index (BSI) is used for semi-quantitative analyses while evaluating bone images [9, 10]. More specifically, the BSI is the ratio of accumulation at sites with a high risk of bone metastasis to the systemic bone mass. Its performance and clinical significance have been widely evaluated with respect to imaging biomarkers for prostate, breast, lung, renal, and other cancers [11–17]. Shintawati et al. found that both the BSI and number of hotspots tend to increase with an increase in uptake duration (i.e., 2-h, 4-h, or 6-h); therefore, they suggested that the uptake time for each patient should be fixed during follow-up or monitoring [18]. In addition, Kaboteh et al. reported a statistically significant increase in the BSI between uptake times of 1 h, 3 h, and 4 h [19]. They also suggested that the uptake time should be standardized if patients are repeatedly monitored using bone scintigraphy. We found that the mean difference in SUVmax in the parietal bones and jawbone lesions was − 0.04 and 3.8%, respectively; therefore, we concluded that the difference in the SUVmax of the 2- and 3-h images was clinically negligible. However, based on the previous BSI-related results, the slight increasing trend observed in the SUVmax of our lesions may not be negligible when images are obtained using uptake times of 4–6 h.

Yamane et al. investigated the quantitative reproducibility of SPECT/CT on different imaging days in 12 patients. The number of days elapsing between the two scans ranged from 4 to 10 days, with a median of 6 days. The results demonstrated strong reproducibility of the SUVmax and peak SUVs, with a strong correlation between these values [20]. Our previous studies also showed high reproducibility of SUVmax values in the same patient sample when evaluating imaging scans of the parietal bones using quantitative bone SPECT on different imaging days [4]. However, compared to the jawbone, parietal bones are less susceptible to infections affecting the dentition.

In the current study, we performed follow-up SPECT (approximately 3 months after the first imaging scan) in three patients. The interval between the imaging sessions was longer than that in the study conducted by Yamane et al. [20]. Nevertheless, on both imaging days, we determined that the SUVmax could be measured just as effectively on scans with 2-h uptake times as on those with 3-h uptake times and that the high reproducibility of these values was maintained between initial and follow-up SPECT. Two representative cases presented in the current study showed almost identical values of accumulation intensities in comparative evaluations of the 2-h and 3-h values for the initial and follow-up scans. Thus, we were able to confirm the high reproducibility of the SUVmax using intra-patient comparisons through a range of approaches in the current study.

### Limitations

Our study had some limitations. First, our study included a relatively small number of enrolled patients and had a single-center cross-sectional study design. A multicenter study with a larger sample size could provide additional information on different imaging protocols. Second, a stand-alone SPECT device was used in this study; therefore, CT-based attenuation correction or scatter correction was not performed, which might have resulted in inferior accuracy of the SUV. Additional research is warranted to determine whether the 2-h uptake time can be further reduced.

### Potential for generalization of results

First, our study was limited to quantitative SPECT findings for the parietal bones and jawbone lesions (each of which is relatively close to the body surface); thus, additional validation is needed to determine if the present results can be generalized and adapted for other bone sites.

Second, the bone tracer used in this study was HMDP, which is known to have a rapid bone uptake time; additional verification is warranted to confirm whether similar results can be obtained for other types of MDP and other bone tracers.

## Conclusions

When a 2-h interval was allotted between the injection of Tc-99 m HMDP and quantitative SPECT imaging of the jawbone, the SUVmax was clinically equivalent to that obtained with an uptake time of 3 h. Thus, an uptake time range of 2–3 h was acceptable; the uptake time can be reduced to only a minimum of 2 h. Consequentially, a 2-h uptake time could be employed to reduce the burden on patients during bone SPECT and reduce the risk of spreading nosocomial infections. Future studies should assess other bone sites further from the body surface to assess the generalizability of the findings.

## Data Availability

The authors have full control of all primary data and have agreed to allow the journal to review the data if requested. The datasets used and/or analyzed during the current study are available from the corresponding author on reasonable request.

## Abbreviations

ARD: anti-resorptive drug
BCF: Becquerel calibration factor
BSI: Bone Scan Index
CI: confidence interval
CT: computed tomography
HMDP: hydroxymethylene diphosphonate
ICC: intraclass correlation coefficient
LOA: limit of agreement
MDP: methylene diphosphonate
MRONJ: medication-related osteonecrosis of the jaw
SPECT: single-photon emission computed tomography
SUVmax: maximum standardized uptake value
Tc-99m: technetium-99 m
